# Study on the Space Charge Characteristics of Polypropylene Insulation Material Under a Polarity Reversal Electric Field

**DOI:** 10.3390/polym17030430

**Published:** 2025-02-06

**Authors:** Xinhua Dong, Guodong Bao, Wei Wang

**Affiliations:** State Key Laboratory of Alternate Electrical Power System with Renewable Energy Sources, North China Electric Power University, Beijing 102206, China; baocckid@163.com (G.B.); wwei@ncepu.edu.cn (W.W.)

**Keywords:** direct current cable, polypropylene, polarity reversal, space charge

## Abstract

High-voltage (HV) cables may experience voltage polarity reversal during power adjustment, leading to the accumulation of space charges inside the insulation material and causing distortion of the internal electric field. To characterize the effect of grafting modification on the insulation properties of polypropylene (PP), various electrical properties were characterized. The results show that grafting modification can significantly improve the electrical properties of PP, with PPG-2 exhibiting the best electrical properties. Compared with PP, the breakdown strength of PPG-2 is increased by 39.27%, and the critical electric field is increased by 36.52%. Meanwhile, the charge accumulation inside the PPG-2 is extremely small after voltage polarity reversal. The mechanism of grafting modification to enhance the electrical properties of PP was explained by analyzing the trap characteristics of the samples. This indicates that grafting modification introduces a large number of deep traps within PP, suppressing the injection and migration of charge carriers. The presence of deep traps weakens the charge accumulation and electric field distortion at the interface. In this paper, the optimal monomer and content of grafted PP were determined, and the insulation properties of the cable under operating conditions were analyzed. The research results offer practical guidance for the development of high-performance grafted PP cable insulation materials and the reliability of cable operation.

## 1. Introduction

High-voltage direct current (HVDC) cable transmission has the advantages of large transmission capacity, space-saving, flexible laying, long transmission distance, and cross-regional interconnection [1,2,3,4]. The transmission capacity of 500 kV HVDC cables has reached 3 GW, so the performance of insulation materials is crucial for the safe and stable operation of the cables. Cross-linked polyethylene (XLPE) is a commonly used insulation material for HV cables due to its excellent electrical properties [5]. However, XLPE is a thermosetting material, and the cable cannot be recycled after retirement. It can only be buried or incinerated, which does not meet the development concept of green environmental protection [6]. At present, scholars have conducted research on thermoplastic recyclable polyolefin materials. Among them, polypropylene (PP), as a thermoplastic insulation material, has become a popular research direction for power cable insulation materials due to its excellent comprehensive performance [7,8].

Compared with XLPE, PP has better electrical properties, does not require a cross-linking process, can be recycled, and has the advantages of high heat resistance and chemical corrosion resistance, making it the best insulation material for new environmentally friendly HV cables [9]. To further improve the insulation properties of PP materials, currently commonly used modification methods include nano-doping [10], organic small molecule compounds [11], grafting modification [12,13], and so forth. Nano-doping can improve the volume resistivity of polymer nanocomposites, inhibit the accumulation of space charge, and increase DC breakdown strength. However, problems such as the agglomeration of nanoparticles and the dissociation and precipitation of organic small molecules may seriously deteriorate the electrical properties of PP and have always been a bottleneck restricting the large-scale production of composite materials. The introduction of organic small molecules, such as voltage stabilizers, significantly improves the breakdown strength of the material, suppresses space charge injection, and greatly improves thermal stability. However, organic small molecules are prone to migrate within the sample and even dissociate directly at high temperatures and high fields, so the long-term stability of organic small molecule-modified composites still needs to be further improved. Grafting modification technology can impart a variety of excellent properties to materials by grafting special monomers onto polymer molecular chains. In recent years, grafting modification technology has been used to improve the electrical properties of PP materials. It was found that PP material grafted with monomers such as maleic anhydride (MAH), styrene (St), 4-acetoxystyrene (AOS), and 4-allyloxy-2-hydroxybenzophenone (AHB) exhibits excellent volume resistivity, space charge suppression ability, and DC breakdown strength [14,15]. Since the graft-modified PP material can significantly improve electrical properties while avoiding the problems of agglomeration of inorganic nanoparticles and migration or even dissociation of organic small molecules, its application in HV cable insulation has shown great potential and has become one of the current research hotspots.

When a power flow reversal occurs in an HVDC transmission system based on a line-commutated converter, the external DC electric field and the distorted electric field caused by space charge accumulation will superimpose on each other, forming a transient electric field with extremely high local field strength inside the insulation of the HVDC cable. This is very likely to exceed the insulation level of the insulation material, resulting in insulation damage to the DC cable. Therefore, the withstand conditions required for cable insulation under polarity reversal conditions are more stringent than those required for DC electric fields [16,17]. However, existing research mainly focuses on the basic electrical properties of grafted PP materials at room temperature, such as breakdown strength, volume resistivity, and space charge characteristics [12,13,14,15]. Existing studies focus more on the insulation properties of the material itself, without considering the influence of operating conditions such as polarity reversal electric fields on the space charge characteristics of PP. This oversight is not conducive to the application of PP in cable insulation. Therefore, it is of great significance to further investigate the space charge characteristics of grafted PP under polar reversal electric fields for the development of high-performance grafted PP insulation materials.

Considering that St is a common grafting monomer for polyolefin materials, it offers the benefits of low cost and easy grafting. Therefore, this article prepared graft-modified PP samples with different grafting contents using St as the grafting monomer [18]. The microstructure and crystal structure of each sample were characterized in detail, and the properties such as breakdown strength, volume resistivity, and space charge under polarity reversal were studied. The experimental results show that, compared with PP, the breakdown strength of PPG-2 is increased by 39.27%, and the critical electric field is increased by 36.52%. Meanwhile, the charge accumulation inside the PPG-2 is extremely small after voltage polarity reversal. Finally, the effect mechanism of grafting St on the electrical properties of PP is discussed in detail. The research results have important reference value for the selection of grafting monomers and the determination of content. Meanwhile, the space charge characteristics of grafted PP under polarity reversal electric fields were analyzed to provide reference for the reliability of cable operation.

## 2. Sample Preparation and Testing Methods

### 2.1. Preparation of Grafted PP Samples

**Figure 1:**
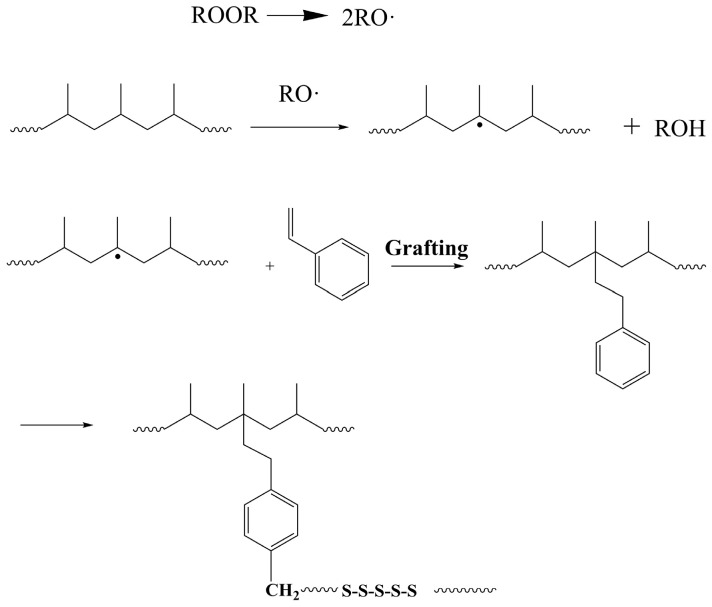
Process of grafting reaction.

St-grafted PP samples were prepared using the melt grafting method. PP (China Petroleum & Chemical Corporation Maoming Branch, Guangdong, China), St (Beijing Bailingway Technology Co., Ltd., Beijing, China), and dicumyl peroxide initiator (Xilong Science Co., Ltd., Guangdong, China) were mixed evenly in a certain ratio, and a single screw extruder was used for reactive extrusion. According to the melting temperature of PP, the extruder was controlled in four stages, within a temperature range of 160 °C–190 °C. The melt grafting reaction mechanism of PP is shown in Figure 1. Based on the current grafting level, the mass fractions of grafted St were determined to be 2.5%, 5%, and 10% [19], and the grafted samples were named PPG-1, PPG-2, and PPG-3, respectively.

Pellets of PP, PPG-1, PPG-2, and PPG-3 were prepared as film samples. The prepared pellets were first heated in a mold at 200 °C for 5 min and then hot-pressed at 15 MPa for 10 min. The mold was subsequently cooled to room temperature under 10 MPa using a liquid-cooling system. Film samples with thicknesses of 100 μm and 200 μm were produced for subsequent testing.

### 2.2. Testing Methods

The grafting effect of St was characterized by Fourier transform infrared spectroscopy (FTIR, Thermo Scientific Fisher Nicolet iS10, USA) to determine whether St was successfully grafted onto PP through a chemical reaction. The testing was performed in transmission mode with a resolution of 4 cm^−1^ and 16 scans. The microstructure of grafted PP was characterized using a scanning electron microscope (SEM, Hitachi SU8000, Japan). The samples were brittle and fractured in liquid nitrogen before testing, and a very thin gold coating was sputtered on the fracture surface to enhance signal intensity. The acceleration voltage of the electron beam was set to 10 kV. The spherulite morphology of each sample was observed using a polarizing microscope (POM, Nikon Eclipse LV100N POL, Japan). The temperature was increased to 200 °C at a rate of 10 °C/min, and then decreased to 135 °C at a rate of 3 °C/min for isothermal crystallization. The melting and crystallization behavior of each sample was studied using differential scanning calorimetry (DSC, TA Instruments Q2000, USA). First, a sample of about 5 mg was heated from 30 °C to 200 °C under a nitrogen atmosphere of 50 mL/min and maintained for 5 min to eliminate the thermal history. Then, the temperature was reduced to 30 °C at a rate of 10 °C/min to obtain the crystallization curve, and subsequently increased to 200 °C at a rate of 10 °C/min to obtain the melting curve. The crystal structure of the samples was characterized by X-ray diffraction (XRD, Bruker D8 Discover, Germany), within the range of 2*θ* = 5–50° and the scanning rate of 3°/min.

The DC breakdown strength of the samples was tested using a breakdown tester (Suzhou Haiwo JN151201, China) equipped with ball-ball electrode. The entire electrode and the sample were placed in silicone oil, and the DC voltage was applied from 0 kV with a ramping rate of 1 kV/s until the sample breakdown. The rated operating temperature of PP cables is generally around 90 °C, and the test temperatures were determined to be 30 °C, 60 °C, and 90 °C [3]. Each sample was tested 16 times, and the experimental data were analyzed by using Weibull distribution. The conduction current of the sample was measured by a standard three-electrode system (Keithley 2635B, USA) with a sample thickness of 100 μm. The test field strength was in the range of 10~60 kV/mm, with a step-up voltage of 10 kV/mm and a test duration of 30 min. The current density of the samples was calculated based on the average value of the current over 25~30 min. The space charge of the samples was tested using the pulse electro-acoustic (PEA) method, with a thickness of 200 μm. The test temperatures were 30 °C, 60 °C, and 90 °C, and the test electric field strength was 40 kV/mm for 60 min. The thermally stimulated depolarization current (TSDC, NovoControl GmbH, Germany) spectra were measured to analyze the trap properties of samples. The samples were first sputtered with gold electrodes and then polarized under 10 kV/mm for 30 min at 90 °C, and subsequently rapidly cooled down to −80 °C. Finally, they were heated up to 127 °C at a rate of 3 °C/min, and the temperature and current signals during the heating process were recorded in real time.

## 3. Results

### 3.1. The Microstructure of St-Grafted PP

**Figure 2:**
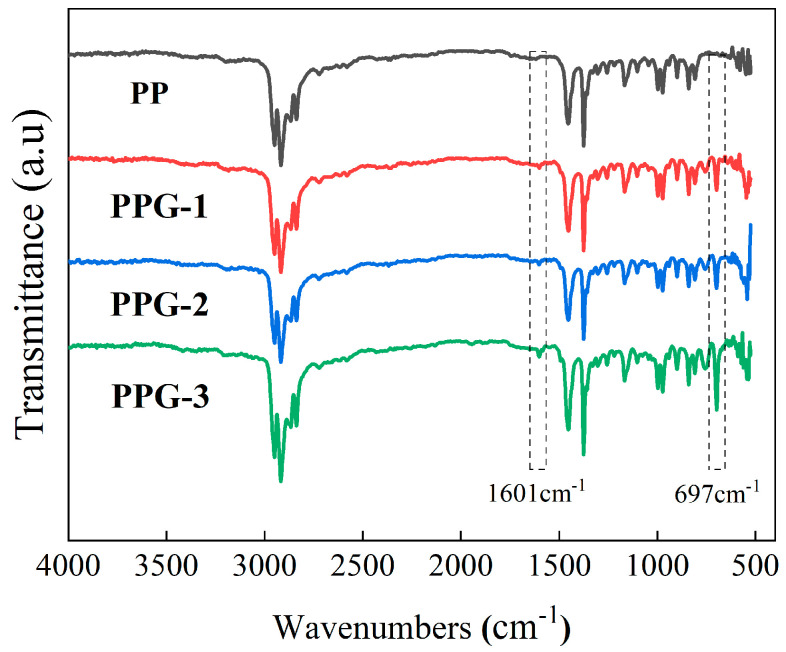
FTIR characterization of the samples.

The FTIR spectra of each sample are shown in Figure 2. It can be observed that, compared with PP, new absorption peaks appear in both PPG samples near 1600 cm^−1^ and 698 cm^−1^. The absorption peaks near 1600 cm^−1^ correspond to the backbone vibration of the benzene ring, and the absorption peaks near 698 cm^−1^ correspond to the C-H out-of-plane vibration of the benzene ring [19]. The intensity of each absorption peak increases with the increase in the St mass fraction, indicating that the St monomer has been successfully grafted onto the PP molecular chain.

**Figure 3:**
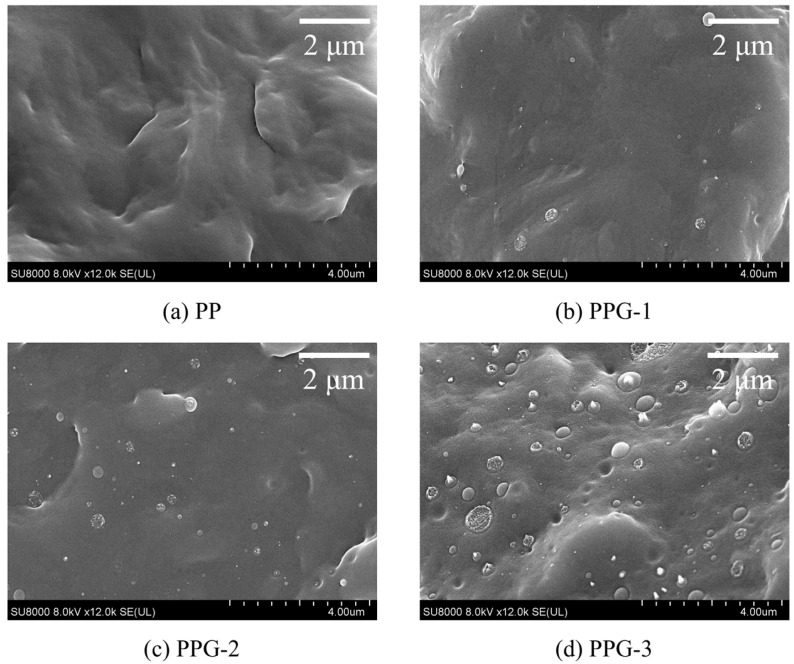
SEM images of the samples.

Figure 3 shows SEM images of the fracture surfaces of each sample. It can be seen that the fracture surface of PP is smooth and flat, and the internal structure is uniform. Spherical structures appeared inside PPG, and the number of spherical structures increased with the increase in grafting content, which may be introduced by grafting the St monomer. This spherical structure changes the microstructure of PP, affects the charge transport process, and thus affects the macroscopic electrical properties.

**Figure 4:**
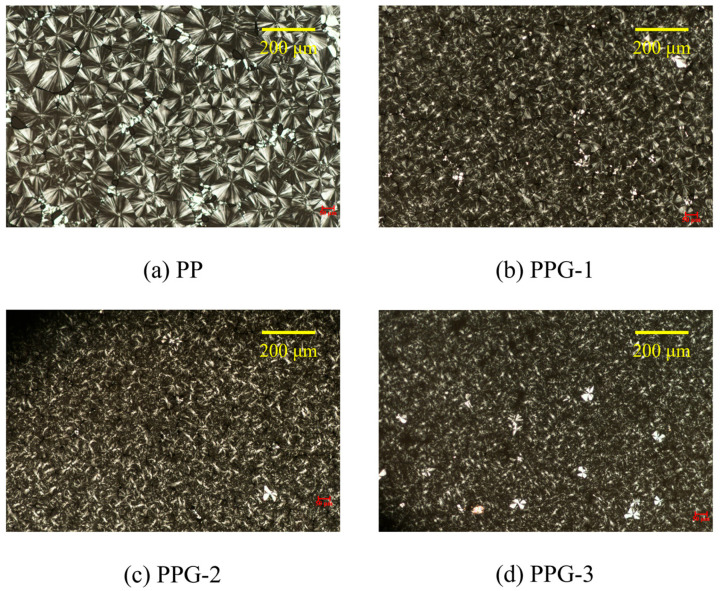
POM images of the samples.

Figure 4 shows the spherulite morphology of each sample after isothermal crystallization. It can be observed that the spherulite size of PP is relatively large, and the boundaries are very clear. After grafting St, the number of spherulites increases, the size decreases, and the boundaries are no longer clear. As the grafting content increases, the spherulites become smaller, and the boundaries disappear more and more. This is due to grafting St, which affects the crystal growth and nucleation process of PP. The St molecular chains are mainly grafted onto the PP molecular chains in the form of side chains, increasing the branching degree of the PP molecular chains and making their arrangement more complex. During the crystallization process, St molecular chains are mostly entangled into a large number of spherical structures due to the repulsion of PP molecular chains. Because the St chains do not crystallize, the spherical structure formed belongs to the amorphous region and increases with the rise in grafting content. In addition, St can induce the heterogeneous nucleation of PP. Therefore, grafting St will lead to an increase in spherulites, smaller diameters, and blurred boundaries, and further strengthen the above trend as the grafting content increases.

**Figure 5:**
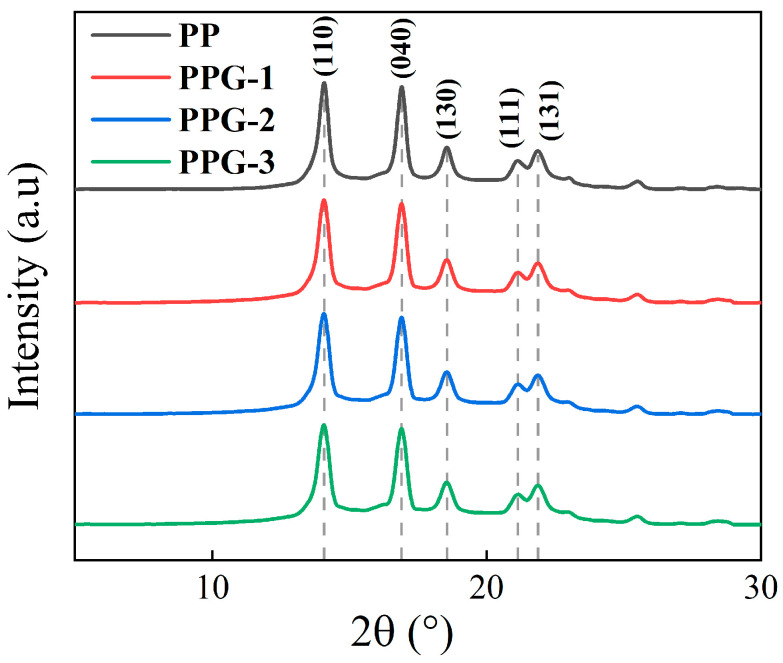
XRD patterns of the samples.

The XRD patterns of each sample are shown in Figure 5. The PP sample exhibits diffraction peaks located at 2*θ* = 14.19°, 16.95°, 18.65°, 21.36°, and 21.88°, respectively, corresponding to the (110), (040), (130), (111), and (131) crystal planes of the *α*-type of PP [20]. Compared with PP, no new diffraction peaks appeared in PPG samples, indicating that grafting modification does not significantly change the crystal structure of PP material. This is because the PP molecular chain is non-polar and incompatible with the St molecular chain, so the crystalline arrangement of the PP molecular chain is basically unaffected.

### 3.2. Thermal Properties of St-Grafted PP

**Figure 6:**
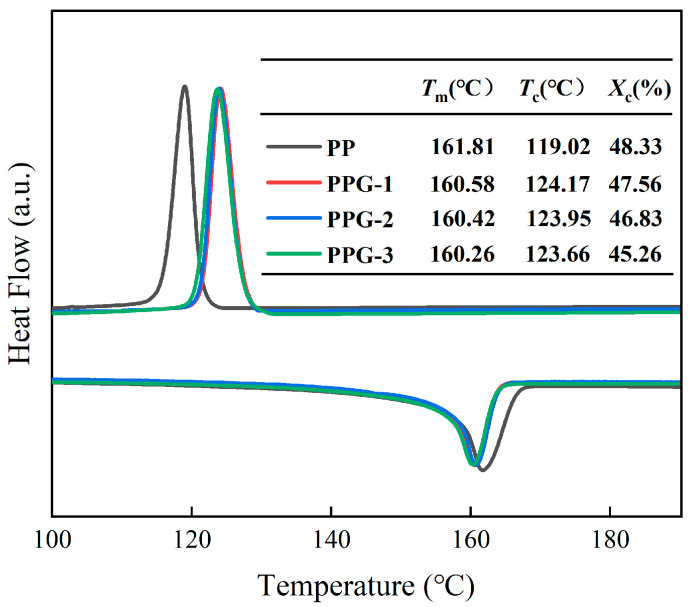
DSC temperature spectra of the samples.

As can be seen from Figure 6, compared with PP, the crystallization temperature of PPG is significantly increased, which is related to the heterogeneous nucleation of the grafting monomer. The crystallization process of polymers is mainly divided into two steps: grain nucleation and growth. For PP materials, the formation of crystal nuclei is mainly homogeneous nucleation, which means that the PP molecular chains in a molten state form orderly chains through thermal movement, becoming crystal nuclei. However, due to the intensification of molecular thermal movement caused by high temperatures, the molecular chains do not easily form an orderly arrangement, making the formation of nuclei difficult.

For the PPG samples, since the grafting monomers are foreign impurities compared to the PP molecular chain, they have a certain probability of adsorbing the PP molecular chains in the melt and arranging them in an orderly manner to form crystal nuclei. Therefore, heterogeneous nucleation can occur at relatively high temperatures, resulting in a significant increase in the crystallization temperature [21]. For PPG samples, the entanglement of grafted side chains significantly inhibits the movement of molecular chains, resulting in a slower grain growth rate, incomplete crystal structure, and lower melting temperature than PP samples [22].

### 3.3. DC Breakdown Strength

**Figure 7:**
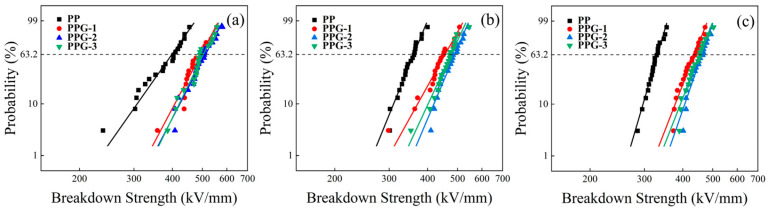
Breakdown strength of the samples under (**a**) 30 °C, (**b**) 60 °C, and (**c**) 90 °C.

Figure 7 shows the DC breakdown strength for each sample at 30 °C, 60 °C, and 90 °C. The breakdown strength was analyzed using the Weibull distribution and calculated according to the following equation [12]:

(1) $$F(E)=1-\exp(-{\left(E/\alpha \right)}^{\beta })$$

where *E* is the breakdown strength of the sample in the experiment, *α* is a scale parameter representing the characteristic breakdown strength at a failure probability of 63.2%, and *β* is a shape parameter that represents the scatter of the measured breakdown strength value.

The breakdown strength parameters of each sample are shown in Table 1, Table 2 and Table 3. It can be observed that PP has the lowest DC breakdown strength, and the DC breakdown strength of PPG is greatly improved at all temperatures. The DC breakdown strength increases and then decreases with the increase in grafting content, with PPG-2 exhibiting the highest DC breakdown strength. As the temperature increases, the DC breakdown strength of each sample decreases to varying degrees. However, at the same temperature, the decrease in breakdown strength of the PPG samples is smaller than that of PP, indicating that the breakdown strength of the PPG samples is less affected by temperature. At 60 °C and 90 °C, with the increase in grafting content, the breakdown strength first increases and then decreases, with PPG-2 still exhibiting the highest DC breakdown strength. At 90 °C, the DC breakdown strength of PPG samples is higher than that of PP at room temperature.

### 3.4. Conductivity

**Figure 8:**
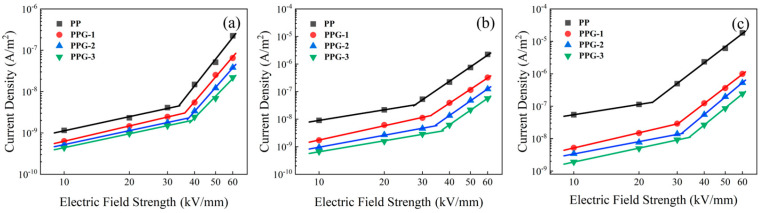
*E*–*J* plots of the samples under (**a**) 30 °C, (**b**) 60 °C, and (**c**) 90 °C.

Figure 8 shows the relationship between the conduction current density and the electric field strength for each sample in double logarithmic coordinates with a segmental fit. The slope is smaller in the first half and larger in the latter part. The smaller slope part is at low field strength, when the current density curve grows slowly. This is because there is less charge injected from the electrode at this time, and most of it is captured by traps. The charges involved in transmission are mainly thermally excited carriers, whose characteristics satisfy Ohm’s law. The larger slope part is under high field strength, where the current density curve grows faster. This is because there is a large amount of charge injected into the electrode at this time, which mainly constitutes the transported charge. Its characteristics satisfy the space charge limited current (SCLC) law [23]. The critical electric field at the turning point is equivalent to the transition of the conductivity mechanism from Ohm′s law to SCLC. The critical electric field of each sample is shown in Table 4.

The results show that grafting St can enhance the critical electric field of PP, and the critical electric field increases with the increase in grafting content. PPG-3 has the highest critical electric field at all temperatures. This indicates that, with more grafting content, more deep traps are introduced, leading to higher trap density and trap level, and consequently, more charge is captured. As the temperature increases, the critical electric field of each sample decreases to varying degrees. This is because the increase in temperature reduces the electrode injection barrier and increases the carrier mobility in the material. However, the threshold field strength of PPG-3 at 90 °C is still higher than that of PP at room temperature.

### 3.5. Space Charge Characteristics

**Figure 9:**
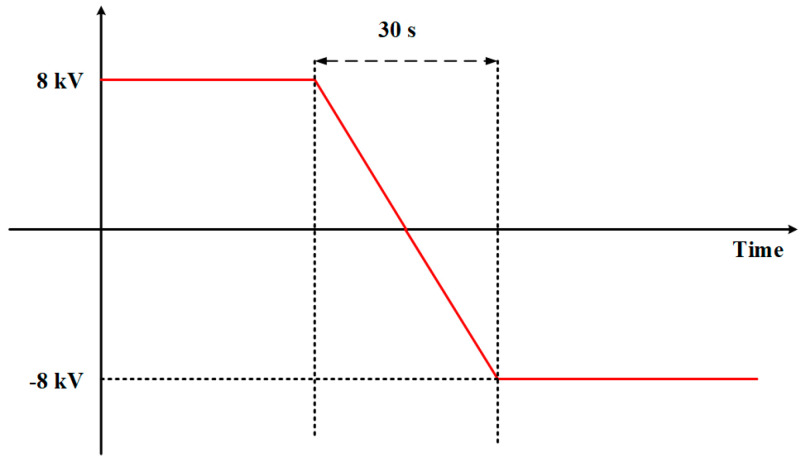
Polarity reversal voltage waveform.

The electric field strength of +40 kV/mm was applied at 30 °C, 60 °C, and 90 °C for 1 h, and the +40 kV/mm was changed to −40 kV/mm at a uniform constant speed within 30 s. The reversal waveforms were maintained for 20 min, as shown in Figure 9, and the space charge waveforms were recorded during this period.

**Figure 10:**
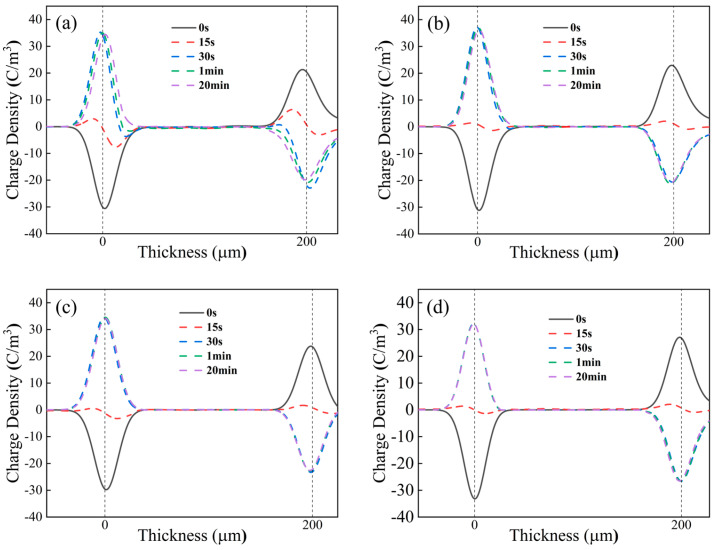
Space charge distribution of (**a**) PP, (**b**) PPG-1, (**c**) PPG-2, and (**d**) PPG-3 under 30 °C.

The space charge distribution of each sample at 30 °C for 30 s of reversal time and 20 min of continued polarization after the completion of reversal is shown in Figure 10. A time of 0 s indicates the space charge distribution at the beginning of the polarity reversal, 15 s indicates the space charge distribution at the moment when the electric field strength drops to 0, 30 s indicates the space charge distribution after the polarity reversal, and 1 min and 20 min indicate the space charge distribution at 1 min and 20 min of polarization after completion of polarity reversal.

It can be seen from Figure 10 that, during the voltage polarity reversal period, the space charge peak at the electrode decreases synchronously as the applied voltage decreases. When the applied voltage changes to negative polarity, the space charge peak at the electrode changes polarity accordingly.

From Figure 10a, it can be seen that, when the voltage polarity reversal is just completed (30 s), there is more heterocharge near the electrode on both sides of the PP sample. At this time, the space charge peak at the electrode shifts outward, indicating a large amount of heterocharge accumulation. The source of this heterocharge is the homocharge injected into the electrode before the polarity reversal, which transforms into heterocharge after the polarity reversal. Due to the reversal occurring before the injection of more homocharge and the completion of the reversal (30 s), the newly injected charge on the electrode cannot be completely neutralized, resulting in the existence of heterocharge. Even after 1 min of complete reversal, there is still some heterocharge, which can cause severe distortion of the nearby electric field and adversely affect the insulation. After the polarity reversal, as voltage continues to be applied, homocharge is rapidly injected at the electrode. The phenomenon of charge peak shifting inward and the peak value falling also occurs. When reaching the steady state, the waveform of the space charge distribution and the steady state waveform before the reversal are roughly a “mirror image” distribution.

For the PPG-1 sample, due to the small amount of homocharge near the electrode before reversal, the amount of heterocharge decreases when the voltage polarity reversal is just completed, and is almost completely neutralized at 1 min. In the PPG-2 and PPG-3 samples, there was almost no heterocharge near the electrode when the voltage polarity reversal was just completed. The injection and accumulation of homocharge after the reversal still weakened with the rise in grafting content. The space charge accumulated inside PPG-3 is the least.

**Figure 11:**
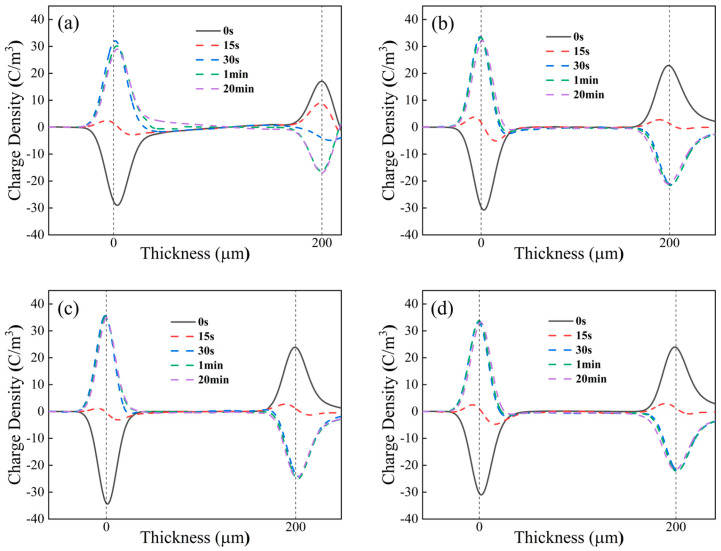
Space charge distribution of (**a**) PP, (**b**) PPG-1, (**c**) PPG-2, and (**d**) PPG-3 under 60 °C.

The space charge distribution of each sample at 60 °C, for a reversal time of 30 s and a continuation of polarization for 20 min after the completion of reversal, is shown in Figure 11.

Comparing Figure 10 and Figure 11, it can be observed that a large amount of homocharge accumulates inside the PP sample when the temperature rises to 60 °C. After the reversal of voltage polarity, with the continuous injection of homocharge, a large amount of homocharge is injected at 20 min, similar to before the reversal. The charge accumulates throughout the entire sample, and the waveform shows a roughly “mirror image” distribution.

For PPG samples, as the temperature increases, the amount of heterocharge increases at the end of the reversal (30 s). However, compared with PP, the amount of homocharge accumulated before reversal is less, so the amount of heterocharge accumulated after reversal is also less, and the amount of heterocharge decreases with the increase in grafting content. PPG-3 has the least amount of heterocharge and the least injection of homocharge after polarity reversal, resulting in the best suppression effect on space charge.

**Figure 12:**
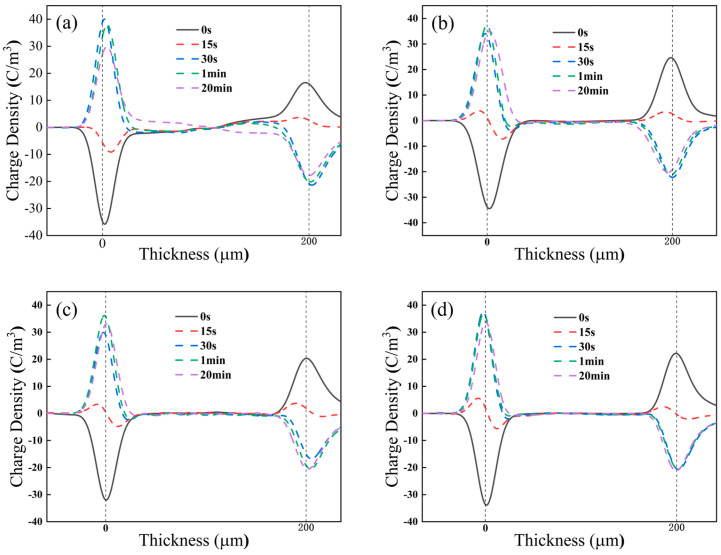
Space charge distribution of (**a**) PP, (**b**) PPG-1, (**c**) PPG-2, and (**d**) PPG-3 under 90 °C.

The space charge distribution of each sample at 90 °C, for a reversal time of 30 s and a continuation of polarization for 20 min after the completion of reversal, is shown in Figure 12.

As shown in Figure 12, it can be observed that, due to the increase in the accumulation of homocharge in the sample before polarity reversal, the amount of heterocharge in the sample when PP just completed polarity reversal at 90 °C is greater than that at 60 °C, which leads to the enhancement of the electric field distortion. However, comparing different PPG samples, it can be seen that, as the grafting content increases, the amount of heterocharge decreases after the reversal due to the improvement in the homocharge accumulation before the polarity reversal. During the subsequent pressurization process, the injection inhibition effect of homocharge still increased with the increase in the grafting content, resulting in PPG-3 still exhibiting the best space charge suppression effect at 90 °C.

### 3.6. Trap Distribution

**Figure 13:**
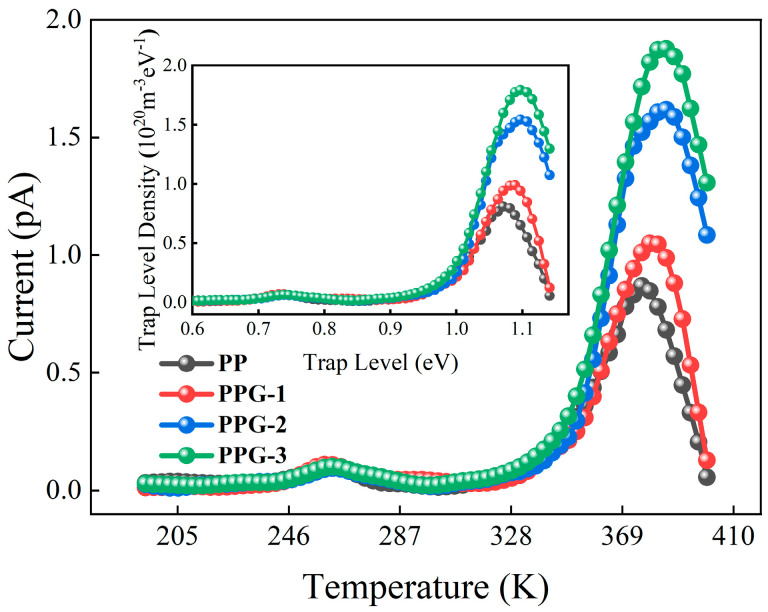
Trap distribution of the samples.

The TSDC curves of each sample are shown in Figure 13. It can be found that the peak current of PP is the smallest, and the temperature of the peak is the lowest. Compared with PP, the current peak temperature of the PPG samples increases, and with the increase in grafting content, the current peak also increases, with PPG-3 having the highest current peak. In order to analyze the trap characteristics of each sample, the trap level distribution of each sample was calculated based on the measurement results of TSDC [24,25], as shown in Figure 13.

Table 5 lists the trap level and trap density for each sample. It can be seen that the trap level of the PPG samples increases and the trap density also increases, indicating that deep traps are indeed introduced. With the increase in grafting content, higher levels and a greater number of traps are introduced. PPG-2 and PPG-3 have the highest trap level of 1.10 eV, exceeding that of PP at 1.07 eV. PPG-3 has the highest trap density of 1.79 × 10^20^ m^−3^eV^−1^, which is more than twice that of PP.

## 4. Discussion

In terms of traps, after grafting St, the electronic band structure of the PPG sample changes significantly, introducing deep traps and increasing trap level and trap density. Since PP mainly injects homocharge, it decreases the electric field strength at the surface, increases the injection barrier, and hinders the injection of charges into the material. The performance in terms of conductivity is reflected in the increase in critical electric field [19].

The increase in temperature decreases the injection barrier and increases the carrier migration rate. Although the space charge injection and accumulation in the PPG samples are higher than that at room temperature, the accumulation is significantly improved compared to that in PP at the same temperature. As the grafting content increases, the inhibition effect on space charge injection and accumulation increases. Although the carrier migration rate and charge injection increases, the trap density and trap level of PPG samples are higher, resulting in better charge suppression in PPG than in PP. Compared with nano-doping, grafting modification enhances the suppression effect of internal space charge in PP. The electrical properties of graft-modified PP are superior to those of nano-doping, which can significantly improve the operational stability and long-term service life of HVDC cables [10].

Under a polarity reversal electric field, the space charges characteristics are mainly determined by the accumulation of space charges before reversal. The more homocharge that accumulates before reversal, the slower the neutralization and dissipation speed after reversal, and the more heterocharge is converted, resulting in greater electric field distortion. Therefore, the better the suppression effect on the accumulation of homocharge before reversal, the better the performance in terms of space charges under the polarity reversal electric field.

From a microscopic perspective, the crystal structure of PP is dense, the amorphous region is loose, and the resistance of the crystal region is greater than that of the amorphous region, resulting in charge mainly being transported in the amorphous region or at the interface. Due to the increase in the number and size of internal spherulites in PPG samples, the charge transport channels become tortuous and narrow, reducing their mobility. From the perspective of traps, grafting St introduces deep traps, and both trap density and trap level increase, which reduces the process of charge trapping and de-trapping, minimizes damage to the molecular chain, and inhibits the initiation of low-density regions. Although PPG-3 has excellent space charge suppression performance, the large amount of St grafting content causes the large size of the spherical structure formed by entanglement during crystallization. This introduces defects in the material, resulting in a lower DC breakdown strength compared to PPG-2.

## 5. Conclusions

The space charge characteristics of PP insulation material under a polarity reversal electric field were studied. The influence mechanism of grafting modification on the microstructure and electrical properties of materials was systematically analyzed, and the optimal grafting content for PP insulation performance was determined. The main conclusions are as follows:

(1) Grafting St introduces grafted side chains into the sample and entangles them with each other, presenting a spherical structure in the fracture surface, with chain breakage observed inside some spherical structures. Grafting modification does not cause changes in the crystal form of PP, but it leads to an increase in crystallization temperature and a decrease in melting temperature and crystallinity.
(2) Grafting modification can significantly improve the electrical properties of PP, with PPG-2 having the optimal DC breakdown strength at all temperatures, showing a maximum increase of 35.86% compared with PP. PPG-3 has the highest critical electric field at all temperatures, with a maximum of 38.7 kV/mm at 30 °C.
(3) Compared with PP, PPG samples effectively inhibit the injection of space charge. Before the voltage polarity reversal, a large amount of homocharge accumulates inside the PP, resulting in poor space charge performance after reversal. The PPG samples effectively improve the situation, and the suppression of space charge gradually increases with the rise in grafting content. PPG-3 has the strongest suppression of space charge under the polarity reversal electric field.
(4) Further research was conducted on the trap characteristics of PPG samples, indicating that the grafted St monomers introduce a large number of deep traps. These deep traps inhibit carrier injection and migration, thereby improving the insulation properties of the material. The heterogeneous nucleation effect of the grafted side chains in the PPG samples increases the number of spherulites in the material, reduces the boundary size, hinders the migration of carriers, and reduces the accumulation of internal charges. Meanwhile, the entanglement of the grafted side chains inside the PPG samples enhances the structural stability of the material and reduces the formation of low-density regions, which improves the insulation properties of PP.

The research results indicate that grafting modification can effectively improve the insulation and high-temperature electrical properties of PP materials. Grafting modification greatly weakens the space charge accumulation in PP under polarity reversal electric fields and enhances the operational stability of cables. It avoids the agglomeration problem that exists in modification methods such as nano-doping and demonstrates excellent development potential. This study demonstrates the feasibility of using grafted PP for HVDC applications, offering insights for scalable production. The research results have important reference value for the selection of grafting monomers and the determination of content. Future work should focus on optimizing grafting techniques for cost efficiency and long-term stability.

## Figures and Tables

**Table 1:** DC breakdown strength of each sample at 30 °C.

Sample	*α* (kV/mm)	*β*	Increase in Breakdown Strength (%)
PP	398	8.69	/
PPG-1	495	11.50	24.37
PPG-2	511	11.75	28.39
PPG-3	503	12.55	26.38

**Table 2:** DC breakdown strength of each sample at 60 °C.

Sample	*α* (kV/mm)	*β*	Increase in Breakdown Strength (%)
PP	361	14.98	/
PPG-1	456	10.99	26.32
PPG-2	490	14.42	35.73
PPG-3	477	13.12	32.13

**Table 3:** DC breakdown strength of each sample at 90 °C.

Sample	α (kV/mm)	*β*	Increase in Breakdown Strength (%)
PP	331	20.77	/
PPG-1	436	15.68	31.72
PPG-2	461	17.60	39.27
PPG-3	453	15.55	35.86

**Table 4:** Critical electric field of each sample at different temperatures.

Critical Electric Field (kV/mm)	30 °C	60 °C	90 °C
PP	33.9	28.1	23.0
PPG-1	36.1	32.8	30.2
PPG-2	37.8	34.0	31.4
PPG-3	38.7	36.3	34.1

**Table 5:** Trap density and trap level of the samples.

Sample	Trap Density (10^20^ m^−3^eV^−1^)	Trap Level (eV)
PP	0.81	1.07
PPG-1	0.99	1.09
PPG-2	1.54	1.10
PPG-3	1.79	1.10

## Data Availability

Data are contained within the article.
